# The loss of *DLG2* isoform 7/8, but not isoform 2, is critical in advanced staged neuroblastoma

**DOI:** 10.1186/s12935-021-01851-w

**Published:** 2021-03-16

**Authors:** Simon Keane, Tommy Martinsson, Per Kogner, Katarina Ejeskär

**Affiliations:** 1 Translational Medicine, School of Health Sciences, University of Skövde, Skövde, Sweden; 2 Department of Laboratory Medicine, Institute of Biomedicine, Sahlgrenska Academy, University of Gothenburg, Gothenburg, Sweden; 3 Childhood Cancer Research Unit, Department of Women’s and Children’s Health, Karolinska Institutet, Stockholm, Sweden

**Keywords:** Neuroblastoma, DLG, *DLG2*, *LIN7A*, L27, Isoform

## Abstract

**Background:**

Neuroblastoma is a childhood neural crest tumor showing large clinical and genetic heterogeneity, one form displaying 11q-deletion is very aggressive. It has been shown that 11q-deletion results in decreased expression of *DLG2*, a gene residing in the deleted region. *DLG2* has a number of different isoforms with the main difference is the presence or absence of a L27 domain. The L27 domain containing DLG proteins can form complexes with CASK/MPP and LIN7 protein family members, which will control cell polarity and signaling.

**Methods:**

We evaluated the DLG gene family and the LIN7 gene family for their expression in differently INSS staged neuroblastoma from publically available data and primary tumors, we included two distinct *DLG1* and *DLG2* N-terminal transcript isoforms encoding L27 domains for their expression. Functionality of *DLG2* isoforms and of *LIN7A* were evaluated in the 11q-deleted neuroblastoma cell line SKNAS.

**Results:**

In neuroblastoma only two *DLG2* isoforms were expressed: isoform 2 and isoform 7/8. Using the array data we could determine that higher expression of DLG members that contain L27 domains correlated to better survival and prognosis. Whilst *DLG1* showed a decrease in both isoforms with increased INSS stage, only the full length L27 containing *DLG2* transcripts *DLG2-isoform 7/8* showed a decrease in expression in high stage neuroblastoma. We could show that the protein encoded by *DLG2-isoform 7* could bind to LIN7A, and increased *DLG2-isoform 7* gene expression increased the expression of *LIN7A*, this reduced neuroblastoma cell proliferation and viability, with increased *BAX/BCL2* ratio indicating increased apoptosis.

**Conclusion:**

We have provided evidence that gene expression of the L27 domain containing *DLG2-isoform 7/8* but not L27 domain lacking *DLG2-isoform 2* is disrupted in neuroblastoma, in particular in the aggressive subsets of tumors. The presence of the complete L27 domain allows for the binding to *LIN7A,* which will control cell polarity and signaling, thus affecting cancer cell viability.

## Background

Neuroblastoma (NB) is a transient embryonic neural crest pediatric tumor with development in the autonomous nervous system, in young children it is one of the most common form of extra cranial solid tumor [1]. The common genetic alterations that occur in aggressive (Stage 4) NB is the deletion of a segment of chromosome region 11q or amplification of the oncogene *MYCN* [2, 3]. Within the 11q-deleted region, resides the gene Discs Large Homologue 2 (*DLG2).* Low expression of *DLG2* is seen in a majority of aggressive NBs, including both the 11q-deleted subset and those with *MYCN* amplification [4]. Also Anaplastic Lymphoma Kinase (ALK) activity seems to affect the *DLG2* expression in NB [5]. Low *DLG2* level forces cell cycle progression [4] and results in an undifferentiated state in NB cells [5]. In addition to NB, abnormally low *DLG2* expression is reported in osteosarcoma [6] and ovarian cancer [7].

**Fig. 1:**
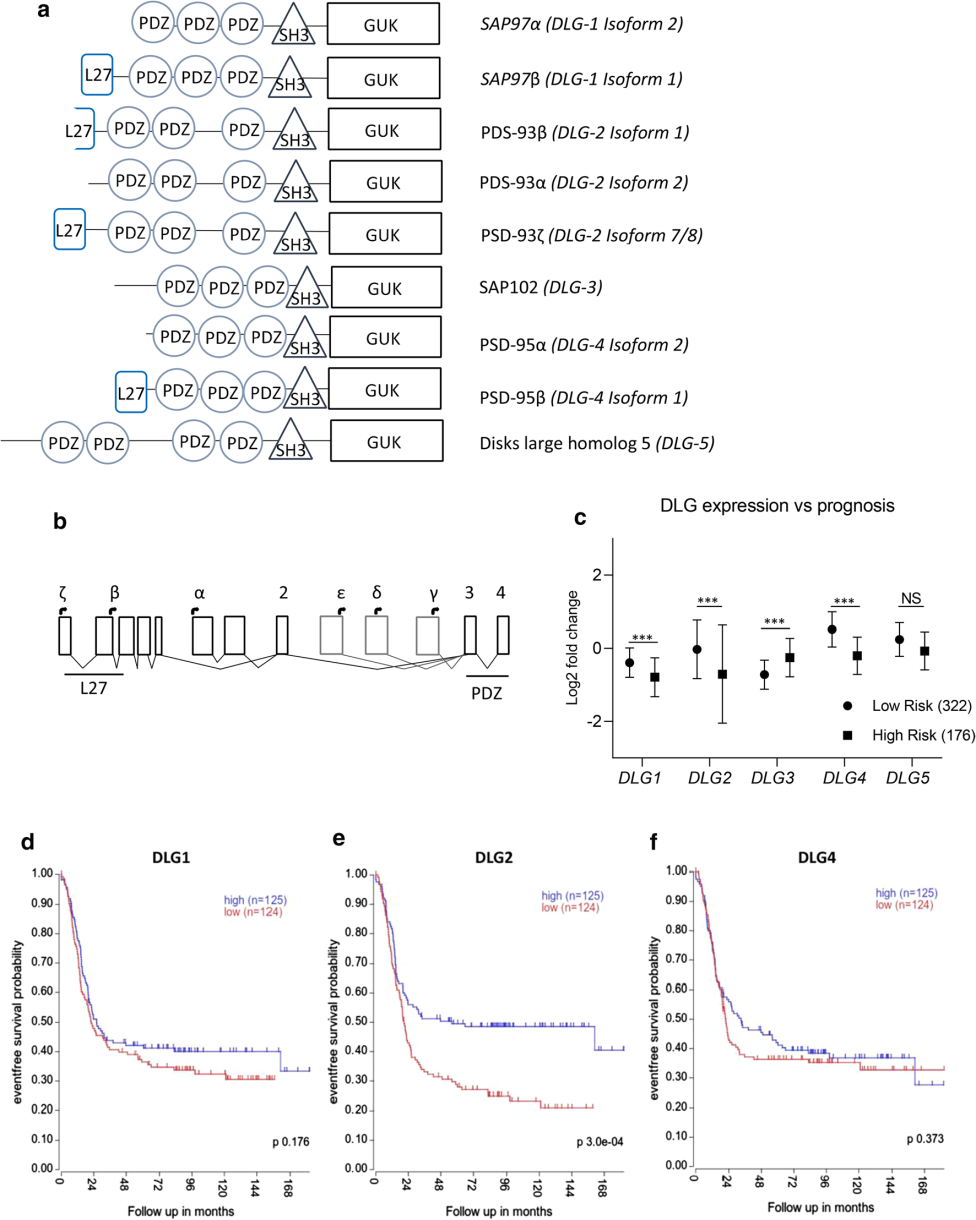
Domains found in DLG-encoded proteins, exon structure of *DLG2* and *DLG* expression in NB.** a** The different isoforms of the DLG family with DLG1, *DLG2* and *DLG4* showing an isoform with *L27* domain. The unique PSD93β protein, encoding just a partial L27 domain. The alpha isoforms of DLG1, DLG2 and DLG4 do not contain the L27 domains and thus have similar structures to DLG3. **b** The exon structure of *DLG2* showing the 5 exons that make up the L27 and linker region in PSD93ζ with mutually exclusive initiation exons for PSD93α. Isoforms PSD93ε, PSD93δ and PSD93γ all have their initiation site after the common exon 2. Transcription start of *DLG2* protein isoform indicated at the top, and protein domains at the bottom. **c** Gene expression of the various DLG family members showing prognosis for 586 patients from the online microarray data with the NB patient dataset (GSE49710) obtained from the R2 Genomics Analysis and Visualization Platform (http://r2.amc.nl). Kaplan–Meier diagrams with a median cut-off showing event free survival with high (blue) vs low (red) expression of (**d)** *DLG1*, (**e**) *DLG2* and (**f**) *DLG4*. The expression data are presented as centered log2 fold change and plotted as mean ± SD. ***p < 0.001

Currently, five members of the *DLG* gene family are identified in human; *DLG1-5*. The DLG gene family members are important in maintaining; cellular structure, polarity and, growth behavior [8–10]. These are achieved by interactions with signaling complexes, protein trafficking to the cellular surface, as well as in supramolecular adhesion [11]. The DLG protein family have a minimum of three PDZ domains, a SH3 domain and a guanylate kinase (GUK) domain (Fig. 1a).

*DLG1, DLG2* and *DLG4* each have various transcription isoforms that either contain or lack a complete “Lin2, Lin7” (L27) domain. The L27 domain is most closely associated with the assembly of signaling complexes and cell polarity complexes [12] by localizing to tight junctions [13], which is important for cell architecture and growth signaling in all cells, including cancer cells. The DLG proteins that lack the L27 domain are designated as the α-protein and contain N-terminal palmitoylated cysteines, derived from two codons that are mutually exclusive to the β-protein [14]. The DLG isoforms that contain the L27 domain are designated as the β-protein with the exception of *DLG2* (Fig. 1a). *DLG1* and *DLG4* [14] have 2 exons encoding the L27 domain whereas *DLG2* (encoding the protein PSD93) has 5 exons encoding the L27 region and SH3 linker region. The currently accepted PSD93β protein is *DLG2* isoform 1 which does not include exon 1 or the start of exon 2, yet follows the standard exon structure of *DLG1* and *DLG4*. This discrepancy was highlighted by Parker et. al. in 2004 [15], where they showed that isoforms 7 and 8 (encoding PSD-93ζ) are the full length protein containing the first three exons resulting in a complete L27 domain. It has even been suggested that it should be renamed as PSD93β [16]. The difference between isoforms 7 and 8 is a single codon with isoform 7 the longer of the two. The *DLG2* isoform 2 encoded protein PSD93α, has no L27 domain and has a separate initiation site at exon 6 encoding the palmitoylated cysteines (Fig. 1b).

The DLG transcripts with the complete L27 domain can form L27 tripartite complexes [17], the L27 mediated protein interaction is often an interaction with three proteins forming a complex of four L27 domains [13, 18]. For the tripartite protein complex to form, a protein with two L27 domains such as Membrane Palmitoylated Protein (MPP) or Calcium/Calmodulin Dependent Serine Protein Kinase (CASK) are required first, subsequently the second L27 domain is provided by the LIN7 family with the final L27 domain provided by the DLG family [19]. The LIN7 family consists of three members, Lin7 Homolog A (LIN7A), LIN7B, and LIN7C; each containing a L27 domain and a PDZ domain. The L27 domain has been shown to direct protein binding so that the resulting complex is diverse and does not contain homodimerization [19], which is otherwise common within the broader membrane-associated guanylate kinase (MAGUK) superfamily of which the DLGs are members.

The DLG isoforms lacking the L27 domains have N-terminal palmitoylated cysteines that target to synapses [14] and increase synaptic strength [14, 20, 21]. All *DLG3* and *DLG5* encoded proteins lack the L27 domain and N-terminal cysteines (Fig. 1a). The *DLG3* encoded protein SAP102 is regulated by the SH3 and GUK domain and is often found in immature neurons, suggesting a specific role in neuron growth and development [22]. Overexpression of *DLG3* results in a loss of adhesion properties in esophageal cells [23] and decreased survival in breast cancer [24]. *DLG5* has been shown to be lost in breast cancer cells [25] with restoration of *DLG5* expression inhibiting cell migration and proliferation [10].

In light of previous studies showing intriguing importance of *DLG2*-expression in NB [4, 5], we have in this study evaluated the expression of all DLGs and its different isoforms, especially considering the L27-domain containing DLG-isoforms, and the important L27 containing interaction partner LIN7A in NB. We have evaluated the different isoforms of *DLG2* in detail and how they relate to the tripartite complex and NB cell viability.

## Methods

### Gene expression analysis

Data for analyses and comparison of *DLG1-5* expression between the different patient subgroups was imported from the R2 platform (http://r2.amc.nl). The independent NB primary datasets; (1): SEQC GSE49710 (microarray), (2): Versteeg GSE16476 (microarray), and (3): Neuroblastoma NCI TARGET data (RNA-seq) were used. The NCI TARGET data was generated by the Therapeutically Applicable Research to Generate Effective Treatments (https://ocg.cancer.gov/programs/target) initiative, phs000218, and the data used for this analysis are available at https://portal.gdc.cancer.gov/projects. RNA from NB cells (SKNAS) and from 22 fresh frozen primary NB samples, staged according to the International Neuroblastoma Staging System (INSS); 5 stage 1–2, 9 stage 3 and 8 stage 4 tumors; were extracted using RNeasy Kit^®^ (Qiagen) according to manufacturer’s protocol. RNA was quantified by NanoDrop (NanoDrop Technologies) and 2 µg of RNA was reverse-transcribed into double stranded cDNA using the High Capacity cDNA Reverse Transcription kit (Applied Biosystems), reaction performed on a T-professional Basic Gradient thermal cycler (Biometra). cDNA corresponding to 20 ng of RNA was used for each qPCR reaction. Taqman Gene probes DLG2 (Hs00265843_m1), LIN7A (Hs00190574_m1), BAX (Hs001180269_m1), BCL2 (Hs00608023_m1), ACTB (Hs99999903_m1) GAPDH (Hs02758991_m1) using TaqMan™ Gene Expression Master Mix (4,369,016, Applied Biosystems). Transcript sequences of the isoforms of *DLG2* were obtained by FASTA search with the human cDNA sequence for each gene. Reactions were prepared for each cDNA using the SYBR^®^ Green Master Mix protocol (Applied Biosystems), primers used according to Table 1.

**Table 1:** *DLG2* NCBI reference sequence and PCR primer target sequence

NCBI reference sequence	Isoform	Protein	Forward primer (5′ to 3′)	Reverse primer (5′ to 3′)
NM_001142699.1	Isoform 1, 7 and 8	PSD93β (975aa)	GCACGGAGCAAGAAGGGAT	AGCTTATTCCAAGCTTTGCT
NM_001364.3	Isoform 2	PSD93α (870aa)	GCTCTCACTCAGTGCCTTCA	GTCCGGAGTGCACAGTAACA
NM_001142700.2	Isoform 3	PSD93 (749aa)	TTTGAGTGTTACCAGCTTTCGCT	TTTCTGTCCCATTGACCGGA
NM_001142702.2	Isoform 4	PSD93 (334aa)	TCAGGTTCCGCTAGTGAGTT	AACCGTCGTCACCTAATCCG
NM_001351274.2 NM_001351275.2	Isoform 7 Isoform 8	PSD93ζ (969aa/968aa)	AGAAGACAGATACTGACCGAGC	CACGGAGCAAGAAGGGATGT

### Ethics statement

Primary NB samples were collected for which written or verbal consent was obtained according to the ethical permits approved by the Karolinska University Hospital Research Ethics Committee (Approval No. 2009/1369‐31/1 and 03‐763).

### Cell lines and cell culture

Human NB cell line SKNAS and HEK293 were obtained from ATCC Cell Line Collection. The cell lines were maintained in RPMI 1640 supplemented with 10% FBS, 1% L-Glutamine, 1% HEPES solution and 1% sodium pyruvate. Cells were maintained at 37 °C with 5% CO_2_.

### Plasmids, siRNAs and transfections

DLG2 (NM_001364) and DLG2 (NM_001351274.2) overexpression plasmids on a backbone of pcDNA3.1/C-(K)-DYK (OHu25658D and OHuq102626D respectively) vector were purchased from GenScript. LIN7A (NM_004664) over expression plasmid on a backbone of pCMV6-AC-GFP (PS100010) was purchased from Origene (RG221902). siRNA targeting *DLG2* (s4122), *LIN7A* (s16836) or Silencer™ Select Negative control No. 1 siRNA (4390843) were purchased from Ambion (Thermo Fischer Scientific). SKNAS and HEK293 cells were grown to 80% confluence and subsequently transfected with; DYK-tagged *DLG2 isoform 7*, DYK-tagged *DLG2 isoform 2*, combined with GFP-tagged *LIN7A*, empty vector “mock” (pCMV6-Ac-GFP), si-*LIN7A* or scrambled negative control “mock”. 100 ng of DNA or 10 pmol siRNA was complexed with 0.3 µl of Lipofectamine 2000 according to the Lipofectamine 2000 reagent forward transfection protocol (Invitrogen; Thermo Fisher Scientific, Inc.).

### Cell growth, proliferation and cell cycle assays

100 µl cell suspension of SKNAS (1 × 10^4^ cells/well) was seeded in 96-well culture plates (Corning Incorporated). After culturing to 80% confluence the supernatant was removed and transfection media was added to the cells. 48 h post transfection, cells were counted using a 60 µm sensor for the Scepter handheld cell counter (Millipore) [26]. Cell proliferation was measured using the MTS/MPS Cell Titer 96^®^ One solution Reagent (Promega) and detecting the color variation (FLUOstar Omega, BMG Labtech) as per the manufacturer’s recommendations. The absorbance values were normalized to the mock transfection and expressed as a percentage. All experiments were repeated three times. Cell cycle analysis was performed using the Cell-clock cell cycle assay (Biocolor). Images were subsequently analyzed using Image J image analysis as per the manufacturer’s instructions. The data presented is the average of three biological replicates. Each experiment series was repeated in triplicate.

### Protein co-immunoprecipitation and Western blot

Protein was extracted from the transfected cells in 6 well plates (1 × 10^5^ cells/well), by aspirating the media and incubating on ice for 5 min then adding ice cold mPER buffer (Thermo Fisher Scientific, 78505). The lysate was co-immunoprecipitated using µMACS isolation kits for DYKDDDDK (Miltenyi Biotech, 130–101-591) and for GFP (Miltenyi Biotech, 130-091-288). Western blot analysis was performed using a Mini-PROTEAN^®^ TGX™ 8–20% gradient gel (BioRad), protein was blotted onto LF-PVDF membrane (8 min, 25 V and 2.5A) using a Trans-Blot^®^ Turbo^™^ Transfer System (BioRad). Blots were subsequently blocked for 1 h in 5% milk in TBST buffer (0.1% Tween-20 and 150 mM NaCl in 10 mM Tris–HCL, pH 7.4) as per the manufacturer’s recommendations. Blots were probed overnight at 4 degrees with antibodies diluted in PBST (0.1% Tween-20 in PBS). Primary antibodies; FLAG tag (FG4R, 1:1000, Invitrogen), LIN7A (PA5-30871, 1:1000, Invitrogen), DLG2 (D4Z4D, 1:1000 Cell Signaling Technology), BAX (2D2, sc-20067, 1:500, Santa Cruz Biotechnology (SCBT)), BCL2 (100, sc-509, 1:500, SCBT) and hFAB Rhodamine Anti-GAPDH (12,004,168, 1:2500, BioRad). The secondary antibodies used were; Starbright goat anti-Rabbit (12,004,161, 1:2500, BioRad), Alexa 488 goat anti mouse (A28175, 1:5000, Invitrogen) and goat anti-mouse Alexa790 (A11357, 1:5000, Invitrogen). All wash stages were 3 × 10 min in 0.1% TBST. Secondary antibodies were incubated for 1 h at room temperature. Image detection was performed on ChemiDoc MP (BioRad).

## Statistical analysis

All data presented are plotted as Tukeys box and whisker plots showing IQR, line at the median, + at the mean with whiskers ± 1.5-fold of interquartile range from at least 3 independent experiments. For all multi-group analyses, differences were determined by one way ANOVA test followed by Holm-Sidak’s multiple comparison test. For comparisons between two groups a Mann–Whitney *U* test was used: ^*^p < 0.05, ^**^p < 0.01, ^***^p < 0.001. All analyses were conducted using GraphPad Prism version 9.0.0 for Windows, (GraphPad Software, www.graphpad.com).

## Results

### Expression of DLG members with L27 domains were inversely correlated to survival and risk

The main difference between different proteins encoded by DLG family members and their isoforms is the presence or absence of an N-terminal L27 domain (Fig. 1a). Unique *DLG2* exons is used to encode the different *DLG2* isoforms, the exon structure and initiation sites of the ζ, β, α, ε, δ and γ protein isoforms is presented in Fig. 1b [15, 16]. We evaluated the association of DLG family expression with event free survival and risk, using online NB patient dataset (GSE49710) and patient dataset (TARGET) obtained from the R2 Genomics Analysis and Visualization Platform (http://r2.amc.nl). Risk stratification showed higher expression in low risk NB for *DLG1* (log2 fc = 0.40, p < 0.001), *DLG2* (log2 fc = 0.68, p < 0.001) and *DLG4* (log2 fc = 0.72, p < 0.001), whereas *DLG3* (log2 fc = − 0.47, p < 0.001) showed lower expression in low risk NB (Fig. 1c). The level of expression of *DLG1* (Fig. 1d) or *DLG4* (Fig. 1f) showed no difference in event free survival whereas high *DLG2* expression was associated with a longer event free survival (p < 0.001) (Fig. 1e).

### DLG2 isoform 7/8 were downregulated in high stage neuroblastoma

**Fig. 2:**
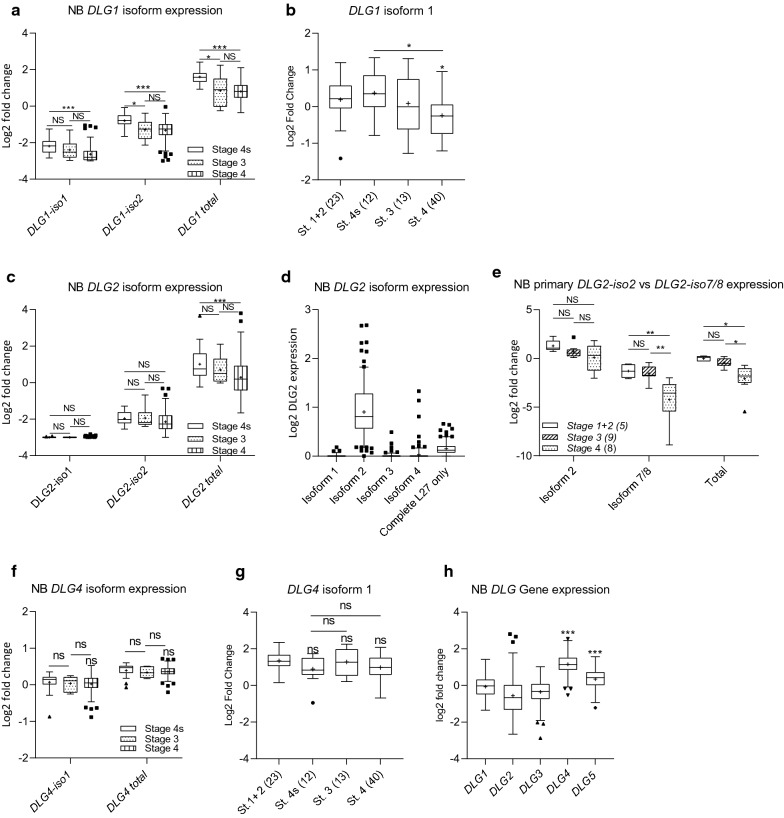
Comparison of DLG family member isoforms by stage. DLG1 isoform and total gene expression by NB stage from (**a**) the NCI TARGET data; phs000218 (RNAseq) and (**b**) NB patient dataset (GSE16476) (microarray). **c** *DLG2* isoform and total gene expression by NB stage from the NCI TARGET data, (**d**) total mean expression level of *DLG2* isoforms in all NB stages, (**e**) qPCR data comparing *DLG2*-isoform 2 and *DLG2*-isoform 7/8 expression in 22 primary NB tumors. DLG4 isoform and total gene expression by NB stage from (**f**) the NCI TARGET data; phs000218 (RNAseq) and (**g**) NB patient dataset (GSE16476) (microarray). **h** comparison of the relative total DLG expression in the NCI TARGET NB dataset. The expression data are presented as median centred log2 fold change and plotted as Tukeys box and whisker plots showing IQR, line at the median, + at the mean with whiskers ± 1.5-fold of interquartile range. Data outside the whiskers are shown as outliers. *p < 0.05, **p < 0.01, ***p < 0.001

We evaluated the expression levels in NB of the L27-domain containing DLG family members, *DLG1*, *DLG2* and *DLG4*, by comparing the total gene expression and transcripts encoding the alpha or beta proteins, using RNAseq-data from the NB patient dataset (TARGET) obtained from the R2 Genomics Analysis and Visualization Platform (http://r2.amc.nl). The data was divided into INSS stage for *DLG1*, *DLG2* and *DLG4*. *DLG1* showed a decreased *DLG1* isoform 1 (*DLG1-iso1)* (ENST00000452595), (encoding SAP97α protein), expression in stage 4 NB compared to the favorable stage 4 s (log2 FC = 0.44, p < 0.001) with no difference between stage 3 and 4 (Fig. 2a). Decreased *DLG1* isoform 2 (*DLG1-iso2)* (ENST00000357674), (encoding L27-containing SAP-97β protein), expression in stage 4 NB compared to stage 4 s (log2 FC = 0.44, p < 0.001) and between stage 3 and stage 4 s (log2 FC = 0.76, p < 0.05), was also seen (Fig. 2a). At the total *DLG1* gene expression level a similar decrease in expression as the *DLG1-iso2* transcript was observed between stage 4 and stage 4 s (log2 FC = 0.80, p < 0.001) and between stage 3 and stage 4 s (log2 FC = 0.76, p < 0.05) (Fig. 2a). We confirmed the *DLG1-iso1* expression by using an independent NB patient dataset (GSE16476) based on microarray data, also showing the similar *DLG1-iso1* expression in the stage 1 + 2 and 4 s tumor groups, both considered low risk tumors (Fig. 2b).

When analyzing the TARGET data, *DLG2* showed no difference in *DLG2* isoform 1 (*DLG2-iso1)* (ENST00000376104), (encoding the truncated L27-containing SAP-93β), expression or *DLG2* isoform 2 (*DLG2-iso2)* (ENST00000398309), (encoding the non-L27-containing PSD-93α), expression when comparing the stages (Fig. 2c). At the total gene expression level (including all *DLG2* isoforms) a decrease in expression was observed between stage 4 and stage 4 s (log2 FC = 0.72, p < 0.001) (Fig. 2c), indicating that isoforms accounting for this difference were not included in this analysis.

We evaluated the expression level of all main *DLG2* isoforms in NB, using the transcript data from the TARGET dataset based off GRCh37. We determined that the *DLG2* isoforms with the highest expression were *DLG2*-*iso2* (ENST00000398309) and *DLG2* L27 only (ENST00000472545), with no or very low expression of isoforms 1 (ENST00000376104), 3 (ENST00000418306) or 4 (ENST00000280241) detected (Fig. 2d). In this chromosome build *DLG2-iso7* or 8 were not included and therefore could not be included in the analysis. The presence of *DLG2* L27 only (ENST00000472545) indicated that isoforms 7/8 were likely expressed, but not captured in this expression data using this chromosome build. Using 22 primary NB samples, we could confirm by qPCR the *DLG2-iso2 *expression observed in the TARGET dataset (Fig. 2e). We could also confirm that there was no expression of isoforms 3 or 4 in our samples. Isoform 1 as a truncated variant of isoforms 7 and 8 (Fig. 1b), could not be uniquely identified by qPCR when compared to isoforms 7/8, and since the isoform 1/7/8 qPCR result showed the same result as the specific isoform 7/8 qPCR, we concluded that isoform 1 was not expressed in our samples (data not shown). No variation in the expression of *DLG2-iso2* (ENST00000398309) was observed between the stages (Fig. 2e), consistent with Fig. 2c. The *DLG2-iso7/8* (ENST00000650630) transcript had decreased expression in the stage 4 tumors when compared to the stage 1 and 2 tumors (log2 FC = 3.1, p < 0.05), reflecting the difference in total *DLG2* expression between differently staged NB (Fig. 2e).

*DLG4* showed no decrease in isoform 1 expression between the stages using the TARGET dataset. Furthermore, there was no change in total *DLG4* expression level between stages (Fig. 2f). We confirmed the isoform expression by using an independent Patient dataset (GSE16476), using microarray data (Fig. 2g).

To evaluate the total DLG gene expression in NB we determined the relative expression of all DLG family members. *DLG1, DLG2* and *DLG3* all showed similar expression levels with *DLG4* and *DLG5* having significantly higher expression (p < 0.001) (Fig. 2h).

### *DLG2* expression correlated to *LIN7* family gene expression and NB samples formed clusters

**Fig. 3.**
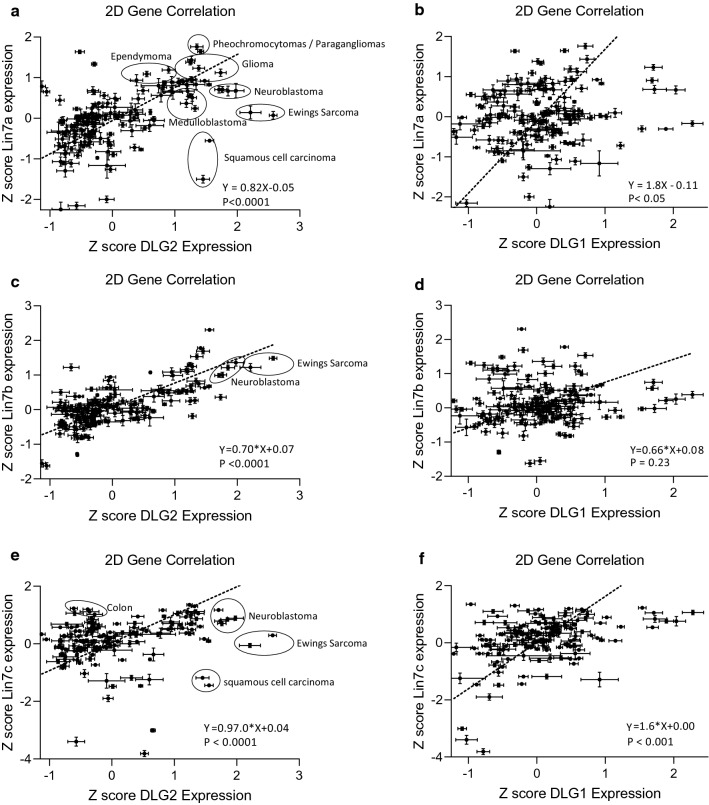
2D gene correlation of *DLG1* and *DLG2* with the *LIN7* family across tumor datasets. Scatter plots with data from 153 available differing tumor datasets sets on the R2 Genomics Analysis and Visualization Platform (http://r2.amc.nl), with *LIN7* expression on the Y-axis and DLG expression on the X-axis using the gene expression mean Z score. **a** *LIN7A* and *DLG2*, **b** *LIN7A* and *DLG1*, **c** *LIN7B* and *DLG2*, **d** *LIN7B* and *DLG1*, **e** *LIN7C* and *DLG2*, **f** *LIN7C* and *DLG1*. The error bars are the standard deviation of the gene expression within the dataset. A line of best fit was created with a Deming (Model II) regression, the 95% confidence interval of the regression is also shown. Clusters were subsequently identified and highlighted

The L27-domain enables binding to other L27-domain containing proteins. An important L27-containing scaffolding protein in signaling complex formation is the LIN7 protein family. The relationship between *DLG2* and *DLG1* gene expression and the various LIN7 binding partners was examined using primary tumor data taken from the Z score of 159 tumor data sets on the R2 Genomics Analysis and Visualization Platform (http://r2.amc.nl). A positive relationship (Y = 0.82x–0.05, p < 0.001) between *DLG2* and *LIN7A* across tumor datasets could be confirmed (Fig. 3a). Clusters were formed based on the spatial coordinates of *DLG2* and *LIN7A* expression. Medulloblastoma (6/7), Ewings sarcoma (2/2), glioma (6/7), pheochromocytomas/paragangliomas (2/2) and NB (5/5) all showed high *DLG2* expression as well as high *LIN7A* expression. The remaining tumors with similar expression included other tumors of the CNS such as glioblastoma, primitive neuroectodermal tumors (PNET) and other brain tumors. Squamous cell carcinoma (2/2) showed high *DLG2* expression with low *LIN7A* expression. The remainder of the tumor dataset, consisting of lung-, colon-, ovarian-, and breast cancers and various lymphomas tended to show low expression of both *DLG2* and *LIN7A* (Fig. 3a). A weak linear relationship could be established between *DLG1* and *LIN7A* (Fig. 3b), however no distinct tumor clusters could be formed. A positive relationship (Y = 0.70x + 0.07, p < 0.0001) could be established between *DLG2* and *LIN7B* across tumor datasets (Fig. 3c). Ewing’s sarcoma (2/2) and NB (5/5) clustered with high *DLG2* expression as well as high *LIN7B* expression. No linear relationship (Y = 0.66x + 0.08, p = 0.23) between *DLG1* and *LIN7B* across tumor datasets could be confirmed (Fig. 3d). A positive relationship (Y = 0.97x + 0.04, p < 0.0001) between *DLG2* and *LIN7C* between tumor datasets could be confirmed (Fig. 3e). Ewings sarcoma (2/2) and NB (5/5) clustered with high *DLG2* expression as well as high *LIN7C* expression. Squamous cell carcinoma (2/2) clustered with high *DLG2* expression and low *LIN7C* expression (Fig. 3e). A positive relationship (Y = 1.6x + 0.00, p < 0.05) between *DLG1* and *LIN7C* across tumor datasets could be confirmed (Fig. 3d), however distinct tumor clusters were not formed.

### *DLG2-isoform 7* expression controlled *LIN7A* expression and the *DLG2-isoform 7* encoded protein could bind to LIN7A

**Fig. 4:**
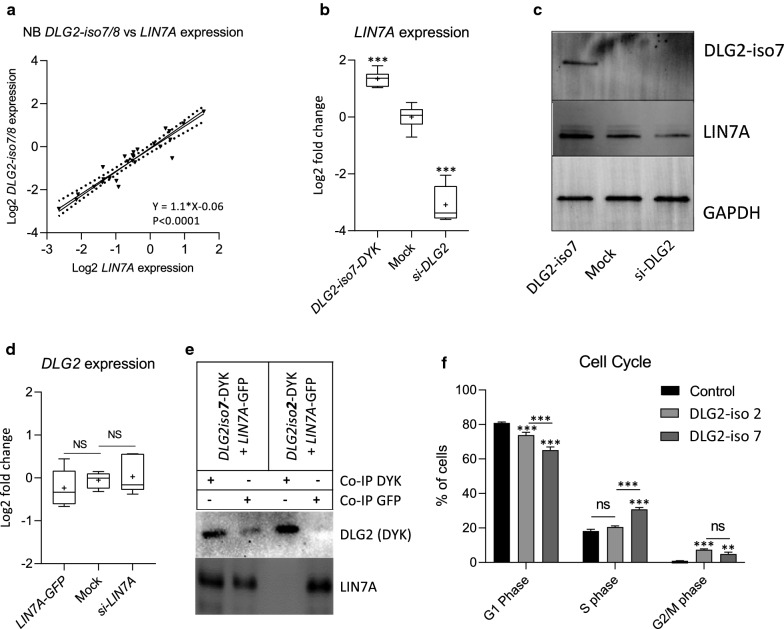
LIN7A expression is affected by *DLG2*-isoform 7. **a** The Relationship of *DLG2*-isoform 7/8 and *LIN7A* expression in 22 NB primary tumor samples. The relative mRNA expression of *DLG2* and *LIN7A* for each sample is determined. The data expressed as relative log2 fold change after normalization to GAPDH and GUSB with linearity determined using a line of best fit, created with a Deming (Model II) regression. **b** LIN7A gene expression 48 h post *DLG2*-isoform 7 over expression (*DLG2-iso7-DYK*) or silencing (si-*DLG2*) in SKNAS cells. **c.** Western blot of SKNAS transfected cells 48 h post *DLG2*-isoform 7 over expression (*DLG2*-iso7-DYK) or silencing (si-*DLG2*) quantifying DLG2, LIN7A and GAPDH expression. **d** *DLG2* gene expression 48 h post *LIN7A* over expression (*LIN7A-GFP*) or silencing (*si-LIN7A*) in SKNAS cells. **e** Co-immunoprecipitation of HEK293 cells co-transfected with *DLG2-iso7-DYK* and *LIN7A-GFP* or *DLG2-iso2-DYK* and *LIN7A-GFP* plasmids. Detection of the lysate with DYK or LIN7A antibody. **f** Cell cycle analysis after *DLG2-iso2 *or *DLG2-iso7* over expression. The data in b and d are shown as the mean ± SD. The data in (**e**–**h**) are presented as Tukeys box and whisker plots showing IQR, line at the median, + at the mean with whiskers ± 1.5-fold of interquartile range ***p < 0.001, ns = not significant

To further evaluate the relationship that was established in Fig. 3a between *DLG2* and *LIN7A* gene expression, we determined the expression of *LIN7A* and *DLG2*-*iso7/8* in NB primary samples. A strong positive correlation (R^2^ = 0.89, Y = 1 0.1x–0.06, p < 0.001) between the expression of *DLG2-iso7/8* and *LIN7A* for 22 primary NB tumors of varying stages was detected (Fig. 4a). To determine if the relationship was causal we over expressed *DLG2-iso7* or knocked down *DLG2* expression by siRNA treatment in SKNAS NB cells. When *DLG2-iso7* was over expressed *LIN7A* expression increased, and *LIN7A* expression decreased following *DLG2* silencing (Fig. 4b). The result was confirmed on protein level by Western blot (Fig. 4c). When *LIN7A* was over expressed or silenced by siRNA we saw no difference in total *DLG2* expression (Fig. 4d). To determine if DLG2-iso7 or DLG2-iso2 bound directly to LIN7A we performed co-immunoprecipitation using co-transfected HEK-293 cells, showing that DLG2-iso7 but not DLG2-iso2 could bind to LIN7A (Fig. 4e). This was expected as DLG2-iso2 lack the L27-domain, and this is the only thing that differs between these two isoforms.

We determined that over expression of either *DLG2-iso2* or *DLG2-iso7* resulted in a decrease in the percentage of cells in G1 phase, as well as an increase in the number of cells in G2/M phase (Fig. 4f). *DLG2-iso7*, but not *DLG2-iso2,* over expression resulted in an increase in the percentage (12.6%, p < 0.001) of cells in S phase when compared to the control (Fig. 4f).

### LIN7A expression was low in high staged tumors and over expression changed the growth behavior of NB cells

**Fig. 5:**
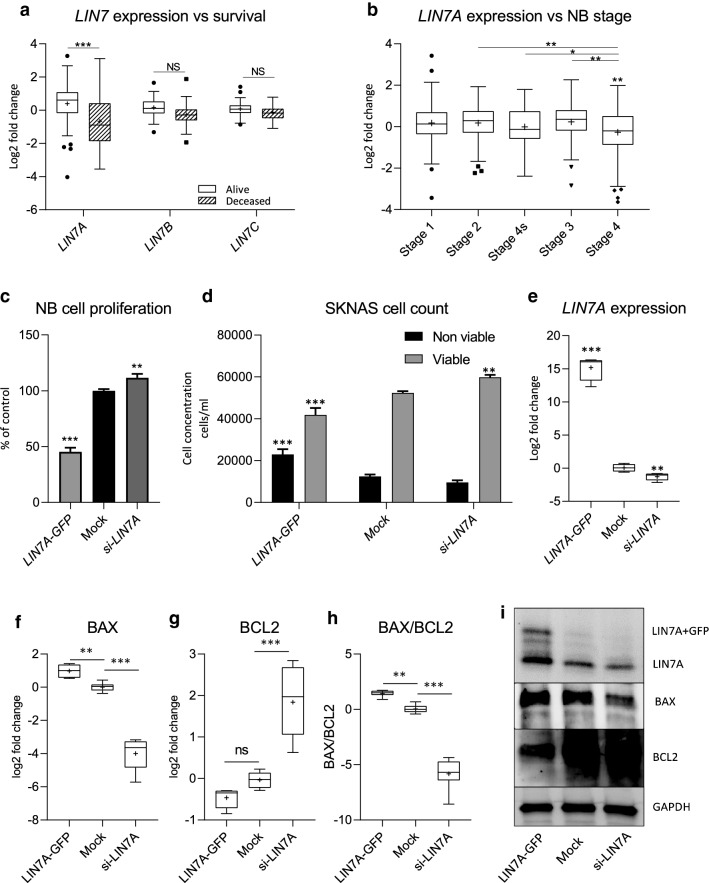
*LIN7A* expression in NB correlates with survival and stage. **a** *LIN7* gene family expression in NB patient dataset (*GSE49710*) stratified by survival (**b**) *LIN7A* gene expression stratified by INSS stage. Cell responses 48 h post *LIN7A* over expression (*LIN7A-GFP*) or silencing (*si-LIN7A*) in SKNAS cells showing: **c** proliferation; **d** viable and non-viable cell fraction. **e** *LIN7A* gene expression analysis 48 h post *LIN7A* over expression (*LIN7A-GFP*) or silencing (*si-LIN7A*) in NB cells (SKNAS). **f** *BAX* **g** *BCL2* and **h** *BAX/BCL2* ratio gene expression, and **i** Western blot showing LIN7A, BAX and BCL2 protein levels, in NB cells after *LIN7A* over expression (*LIN7A-GFP*) or silencing (*si-LIN7A*). The data shown is the pooled average of 3 experiments. The data in c and d are shown as the mean ± SD. The data in (**e**–**h**) are presented as Tukeys box and whisker plots showing IQR, line at the median, + at the mean with whiskers ± 1.5-fold of interquartile range *p < 0.05, **p < 0.01, ***p < 0.001, ns = not significant

To further investigate the importance of *LIN7A* we evaluated the association of LIN family expression with survival and INSS stage, using online microarray data in the NB patient dataset (GSE49710) obtained from the R2 Genomics Analysis and Visualization Platform (http://r2.amc.nl). The data was divided into survival outcome; alive or deceased. *LIN7A* (log2 fc = 1.06, p < 0.001) showed a decrease in expression in the deceased patients compared to the patients that survived (Fig. 5a). *LIN7B* (log2 fc = 0.43, p = 0.09) and *LIN7C* (log2 fc = 0.20, p = 0.66) showed no difference in expression (Fig. 5a). The expression of *LIN7A* was then stratified by INSS stage. Stage 4 tumors showed the lowest expression compared to stage 1 (log2 fc = 0.44, p < 0.01), stage 2 (log2 fc = 0.44, p < 0.001), stage 4 s (log2 fc = 0.25, p < 0.05) and stage 3 (log2 fc = 0.50, p < 0.01) (Fig. 5b). Over expression of *LIN7A* in NB cells (SKNAS) resulted in slower proliferation compared to the control (Fig. 5c, p < 0.001), and we observed a decrease in the number of viable cells (Fig. 5d, p < 0.001) and an increase in the non-viable cell fraction (Fig. 5d, p < 0.001) in cells with increased *LIN7A* expression. *LIN7A* silencing in SKNAS cells resulted in an increase in cell proliferation (Fig. 5c, p < 0.01), with an associated increase in viable cell number, no effect in the non-viable cell number was observed (Fig. 5d). The *LIN7A* over expression after expression plasmid transfection, and *LIN7A* silencing by siRNA treatment of NB cells (SKNAS) was confirmed by qPCR (Fig. 5e). We detected increased gene expression of *BAX* (Fig. 5f), no alteration in *BCL2* gene expression (Fig. 5g) and an increase in the ratio of *BAX/BCL2* (Fig. 5h), indicating an increased level of apoptosis, when *LIN7A* was over expressed. The opposite was seen when *LIN7A* were silenced, then we detected a decrease in *BAX* gene expression (Fig. 5f), increased *BCL2* expression (Fig. 5g) and a decrease in the *BAX/BCL2* ratio (Fig. 5h). This effect could also be confirmed on protein level by Western blot (Fig. 5i).

## Discussion

We have previously established that *DLG2* is a candidate tumor suppressor gene with importance in 11q deleted NB as well as a downregulated target of the oncogene *MYCN*, commonly amplified in aggressive NB [4]. During which, we did not explore the various isoforms of *DLG2* and the effects that the resulting proteins have on NB. As we have shown in Fig. 1a *DLG1*, *DLG2* and *DLG4* all have isoforms that either contain an N-terminal L27 domain or palmitoylated cysteines. When the palmitoylated cysteines are present they modulate homo- or heterodimers with other palmitoylated cysteines that bind synaptic proteins, contributing to the function and strength of the post synaptic density [27]. We could show that there was an overall loss of *DLG1* in the high INSS stage tumors with no difference seen between the α- and β-isoforms (Figs. 1b, 2a). We could also show that *DLG4* isoform expression did not differ in any of the NB stages (Fig. 2c), despite that higher expression correlated with both survival and prognosis (Fig. 1c, f). We showed that *DLG2* displayed differential isoform expression in the high staged tumors (Fig. 2e), the decreased expression of the L27 domain containing *DLG2 isoform 7/8* in the high stage NB (Fig. 2e) highlights the importance of the L27 domain of *DLG2* in NB. Over expression of *DLG2 isoform 7* in NB cells resulted in an increased proportion of cells in S-phase (Fig. 4f), similar to the *BAP1* NB tumor suppressor [28]. This increase in S-phase was not seen after *DLG2 isoform 2* over expression.

The L27 domain is involved in protein interactions, mainly the formation and correct localization of scaffolding and receptor proteins. The localization of L27 domain containing proteins to the membrane bound receptors indicates a signaling regulatory role in these receptors. The formation of the tripartite complexes is known to contain four L27 domains [29], with one protein such as CASK or the MPP family providing two L27 domains and serving as the platform on which the complex is built [29]. The L27 domain containing members of the DLG family have been shown to bind to the N-terminal L27 domain, whereas the LIN family has been shown to bind to the adjacent L27 domain [29]. The presence of the L27 domain is important for the binding of *DLG-*β encoding proteins into this complex. The LIN7 that is present also determines which DLG will likely bind, with *DLG1* encoding proteins and LIN7C showing a strong preference, replicating the already known binding patterns [13, 30]. Whereas, *DLG2* is more of a generalist with expression correlating to all LIN7 homologues (Fig. 3a, c, e), however the clear stratification of tumors seen with *DLG2* and *LIN7A* indicated there may be a causal relationship between the two (Fig. 3a). We were able to show that an increase in *DLG2-iso7* resulted in an increase in *LIN7A* expression (Fig. 4b), but there was no alteration in total *DLG2* expression when *LIN7A* was over expressed (Fig. 4c). Furthermore we could show that *DLG2-iso2* could not bind to LIN7A showing that the L27 domain of *DLG2-iso7* is required for this binding to occur (Fig. 4d). The binding complexes that form as a result of the different L27 containing DLG members will likely have slight functional differences, depending on which base protein is present as well as which DLG and LIN7 family members are bound into the complex, yet have a high degree of redundancy [31]. The various permutations of the base protein, DLG and LIN7 families exponentially expand the different types of the complex that can form.

The depletion of *LIN7A* in neurons has previously been shown to result in abnormal neuronal migration [32], a feature of NB [33]. Clinical cases have also shown that loss of the *LIN7A* loci results in cellular hyperplasia [32]. We were able to replicate these clinical results with the knockdown of *LIN7A* in NB cells, resulting in increased cell number and proliferation, as well as a decreased *BAX/BCL2* ratio indicating decreased level of apoptosis (Fig. 5). The BAX and BCL2 levels are known to be important in regulating apoptosis, particularly in regulating cell differentiation into neurons [34]. However, increased *LIN7A* expression has been previously shown to be associated with a loss of polarity in breast cancer cells [35] as well as increased proliferation in hepatocellular carcinoma [36] and ovarian cancer [37]. Our analysis showed that these previously established tumor types in which *LIN7A* is oncogenic or disruptive tended to cluster with low *DLG2* expression (Fig. 3a). Tissue specificity may account for the altered function observed.

The deletion of 11q in NB is known to be heterozygous and hence leaves one copy of any potential tumor suppressor gene in this region. It has been established that any TSG will probably be involved in a haploinsufficient mechanism due to the general lack of a second hit. Having a gene with two distinct structural isoforms with separate functions resulting in differing protein localization increases the likelihood that *DLG2* may have a haploinsuffient mechanism. The fact that the other members of the DLG family with L27 domains have such a high degree of structural homology, the correct function of the tripartite complex as a whole must be important to the cell. We suggest that whilst there is probably a high degree of redundancy within the DLG family for the tripartite complex function it is most likely highly sensitive to disruptions like 11q deletion or lower expression of another DLG family member.

## Conclusions

We have provided evidence that gene expression of the L27 domain containing *DLG2-isoform 7/8* but not L27 domain lacking *DLG2-isoform 2* is disrupted in NB, in particular in the aggressive subsets of tumors. The presence of the complete L27 domain allows for the binding to *LIN7A,* which will control cell polarity and signaling, thus affecting cancer cell viability.

## Data Availability

The datasets analyzed during the current study are available in the 'R2: Genomics Analysis and Visualization Platform repository, [http://r2.amc.nl]. The datasets analyzed are SEQC GSE49710 (microarray) and Neuroblastoma NCI TARGET data (RNA-Seq). The results generated from the NCI TARGET data was generated by the Therapeutically Applicable Research to Generate Effective Treatments (https://ocg.cancer.gov/programs/target) initiative, phs000218. The data used for this analysis are available at https://portal.gdc.cancer.gov/projects.

## Abbreviations

ALK: Anaplastic lymphoma kinase
CASK: Calcium/Calmodulin Dependent Serine Protein Kinase
*DLG*: Discs large homologue
GUK: Guanylate kinase
INSS: International neuroblastoma staging system
*iso*: Isoform
L27: Lin2, Lin7
LIN7A: Lin7 Homolog A
MAGUK: Membrane-associated guanylate kinase
MPP: Membrane palmitoylated protein
NB: Neuroblastoma
